# Effects of neural estrogen receptor beta deletion on social and mood-related behaviors and underlying mechanisms in male mice

**DOI:** 10.1038/s41598-020-63427-4

**Published:** 2020-04-10

**Authors:** Carlos Dombret, Lydie Naulé, Anne-Charlotte Trouillet, Caroline Parmentier, Hélène Hardin-Pouzet, Sakina Mhaouty-Kodja

**Affiliations:** Sorbonne Université, CNRS, INSERM, Neuroscience Paris Seine – Institut de Biologie Paris Seine, 75005 Paris, France

**Keywords:** Aggression, Social behaviour

## Abstract

Estradiol derived from neural aromatization of testosterone plays a key role in the organization and activation of neural structures underlying male behaviors. This study evaluated the contribution of the estrogen receptor (ER) β in estradiol-induced modulation of social and mood-related behaviors by using mice lacking the *ERβ* gene in the nervous system. Mutant males exhibited reduced social interaction with same-sex congeners and impaired aggressive behavior. They also displayed increased locomotor activity, and reduced or unaffected anxiety-state level in three paradigms. However, when mice were exposed to unescapable stress in the forced swim and tail suspension tests, they spent more time immobile and a reduced time in swimming and climbing. These behavioral alterations were associated with unaffected circadian and restraint stress-induced corticosterone levels, and unchanged number of tryptophan hydroxylase 2-immunoreactive neurons in the dorsal raphe. By contrast, reduced mRNA levels of oxytocin and arginine-vasopressin were observed in the bed nucleus of stria terminalis, whereas no changes were detected in the hypothalamic paraventricular nucleus. The neural ERβ is thus involved to different extent levels in social and mood-related behaviors, with a particular action on oxytocin and arginine-vasopressin signaling pathways of the bed nucleus of stria terminalis, yet the involvement of other brain areas cannot be excluded.

## Introduction

Estradiol derived from neural aromatization of gonadal testosterone by the cytochrome P-450 aromatase contributes to the regulation of several neural functions in male rodents. This regulation starts as early as the perinatal period, to promote masculinization and defeminization of neural structures leading to the expression of male-typical behaviors (olfactory preference, sexual behavior, aggression), and neuroendocrine responses (regulation of the hypothalamus pituitary-gonad axis) in adulthood^1,2^. Estradiol-induced effects in the nervous system are not restricted to reproductive functions and behaviors but extend to several other neural functions including social and mood-related behaviors, or cognition^3,4^.

In the nervous system, estradiol acts mainly through two nuclear estrogen receptors (ER) α and β, with increasing evidence suggesting a potential involvement as well of non-genomic pathways operating through the membrane G protein-coupled ER known as GPER1 or GPR30^5^. In order to decipher the involvement of ERβ in neural estradiol-induced effects, we generated a mouse line lacking *ERβ* in the nervous system^6,7^, by using a transgene which targets Cre recombinase-mediated excision of *ERβ* exon 3 in neuronal and glial progenitors as early as embryonic day 10^8,9^. A first characterization of this mouse line showed that neural ERβ is not involved in the perinatal masculinization and defeminization of male sexual behavior and related brain areas^7^. We thus addressed the question of whether this neural receptor mediates the estradiol-induced modulation of social and mood-related behaviors. Previous studies using global *ERβ* knockout mice reported normal or even increased aggression in the resident-intruder paradigm^10–12^. In contrast, a more recent study conducted by the same group reported that restricted knock-down of *ERβ* gene in the medial preoptic area throughout pubertal and adult stages reduced aggressive behavior in the same behavioral test^13^. No effects were observed when *ERβ* was knocked down in the medial amygdala. A recent reevaluation of global *ERβ* knockout mice led to the conclusion that reduced aggressive behavior of mutant males can be observed in another behavioral test using a neutral testing cage^14^. Furthermore, yet another study on *ERβ* knockout males showed no impairment of social recognition memory^15^.

Concerning mood-related behaviors, few studies have been conducted in male mice and rats. This either resulted in minor effects induced by global *ERβ* knockout in mice^16^, or significant changes following chronic administration of selective ERβ agonists or androgen metabolites acting at ERβ on the anxiety state level in rats^17–19^. By comparison, more studies are available on female behaviors and these all converge on the role of ERβ in mediating estradiol effects^6,16,20–23^. This involves at least in part the maintenance of serotonergic neurons and regulation of the expression of tryptophan hydroxylase (TPH), the key enzyme involved in serotonin synthesis in the dorsal raphe^24^.

In this context, the present work was undertaken in order to evaluate in the same study the neural role of ERβ in social and mood-related behaviors using male mice lacking the *ERβ* gene in the whole nervous system, without interference with peripheral effects of this receptor. For this purpose, control and mutant mice were subjected to behavioral tests that measure social interaction, anxiety-related behavior (elevated O-maze, dark-light box and open field), locomotor activity, despair-like behavior (forced swim and tail suspension tests), sucrose preference test, and aggressive behavior (resident-intruder test). Corticosterone levels were measured under basal circadian and induced stress conditions. The number of TPH2-immunoreactive neurons in the dorsal raphe as well as the expression levels of two neuropeptides involved in the expression of social behaviors i.e. oxytocin (OT) and arginine-vasopressin (AVP) were monitored in key brain areas.

## Results

### Neural *ERβ* deletion interferes with social interaction

**Figure 1:**
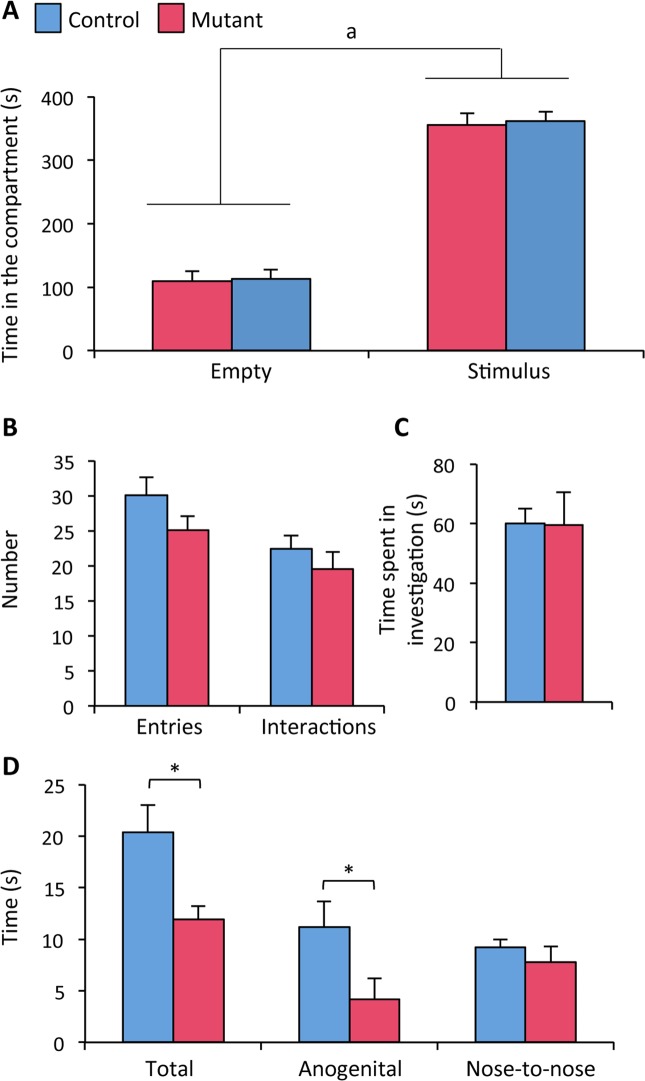
Sociability and social interaction in control (ERβ^fl/fl^) and mutant (ERβ^NesCre^) male mice. (**A–C**) Sociability: Time spent in the empty and stimulus compartments of the three-chamber test (**A**), number of entries and interactions in the stimulus compartment (**B**), and time spent in olfactory investigation of the stimulus (**C**). Data are expressed as means ± S.E.M (n = 12–15 males per genotype). ^b^p < 0.0001: effect of compartment with males more interested in the stimulus compartment. (**D**) Social interaction: Time spent in total, anogenital and nose-to-nose interaction. ^*^p < 0.05 versus the control group.

The impact of neural *ERβ* mutation on sociability was assessed using a three-chamber paradigm containing a compartment with an empty corral and a compartment with a corral containing a mouse stimulus, at either side of the middle chamber. Figure 1A shows no effect of the genotype (F_(1, 25)_ = 0.32, p = 0.58), but an effect of compartment (F_(1, 25)_ = 136.31, p < 0.0001). Males spent more time in the compartment of the male stimulus in comparison to the empty one, regardless of their genotype. The number of entries in the stimulus compartment and number of interactions, as well as the time spent in investigation of the stimulus were not significantly different between controls and mutants (Fig. 1B,C).

Males were then analyzed in a test cage containing clean bedding, for their social interaction with an age- and sex-matched congener, which was free of movement. The total time spent in interaction was significantly reduced for mutant males (−41%) in comparison to their control littermates (Fig. 1D). Detailed analyses of interaction by scoring the time spent in anogenital chemoinvestigation and nose-to-nose interaction showed reduced anogenital investigation (−63% versus controls), and unchanged nose-to-nose interaction in mutant males by comparison to controls.

Therefore, these data show that ERβ^NesCre^ males exhibit normal sociability, but display impaired social interaction in comparison to their control littermates.

### Effects of neural *ERβ* deletion on locomotor activity and mood behaviors

**Figure 2:**
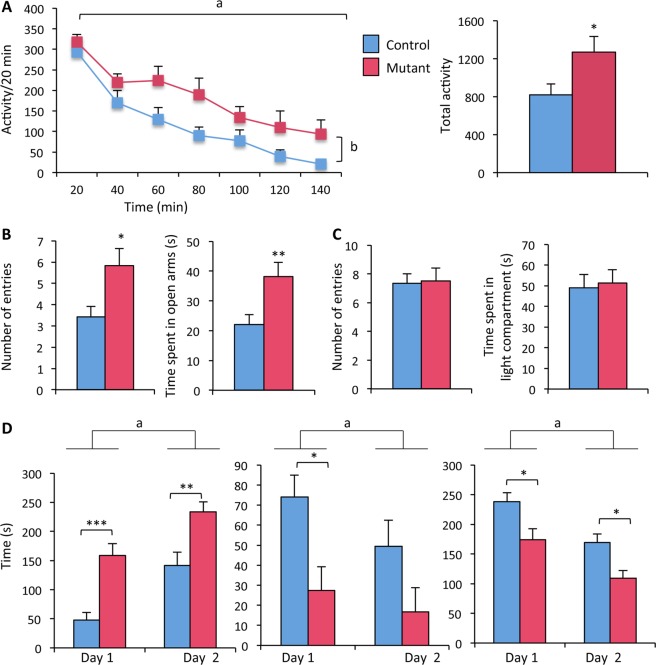
Effects of neural *ERβ* knockout on locomotor activity, anxiety- and despair-like behaviors in male mice. (**A**) *Left* – Locomotor activity per 20 min recorded for males (n = 12–15 males per genotype). ^a^p < 0.001: effect of time; ^b^p < 0.05: effect of genotype with mutants more active than their control littermates. *Right* – Total activity measured during the 140 min of the test. ^*^p < 0.05 versus control littermates. (**B-C**) *Left* – Number of entries in the open arms of the O-maze (**B**), or light compartment of the dark-light box (**C**). *Right* – Time spent in the open arms of the O-maze (**B**), or light compartment of the dark-light box (**C**). (**D**) *Left* – Time spent in immobility over two consecutive days in the forced swim test (n = 12–15 males per genotype). Two-ANOVA shows an effect of time (^a^p < 0.001); post hoc analyses show increased time in immobility for mutants at day 1 (^a^p < 0.001 versus control) and day 2 (^**^p < 0.01 versus control). *Middle* – Time spent in climbing. Two-ANOVA shows an effect of time (^a^p < 0.05); post hoc analyses show reduced time in climbing for mutants at day 1 (^*^p < 0. 05 versus control). *Right* – Time spent in swimming. Two-way ANOVA shows an effect of time (^a^p < 0. 0.001); post hoc analyses show reduced time in swimming for mutants at days 1 and 2 (^*^p < 0. 05 versus control).

Potential effects of neural *ERβ* mutation on locomotor activity were assessed on the same animals examined for social behaviors using a computed circular corridor, where the response to novelty was measured for 140 min (Fig. 2A; Left panel). Two-way ANOVA showed an effect of genotype (F_(1, 25)_ = 5.90, p = 0.02) and time (F_(6, 150)_ = 33.29, p < 0.0001). Analysis of cumulative activity over the 140 min of the test also showed an effect of genotype, with an increased activity of mutant males (+48% over controls, Fig. 2A; Right panel).

The potential effects of neural *ERβ* deletion on anxiety-related behavior were explored using paradigms with different anxiogenic factors. In the elevated O-maze test, the number of entries and time spent in the open arms were increased in mutant males by comparison to their control littermates (+70% and +73% versus controls, respectively, Fig. 2B), suggesting an anxiolytic-like effect of ERβ^NesCre^ mutation. In contrast, no differences were found between the two genotypes in the number of entries and time spent in the light compartment of the dark-light box (Fig. 2C). Males were also analyzed in the open-field test, and the obtained data show a comparable time spent in the central zone, as well as in the periphery, between controls and mutants (Supplemental Fig. 1).

Behavioral despair was analyzed in the forced swim test for 6 min on two consecutive days. The time spent in immobility, climbing or swimming was compared between the two genotypes. Two-way ANOVA showed an effect of the day (F_(1, 25)_ = 49.39, p < 0.0001) and genotype (F_(1, 25)_ = 18.34, p = 0.0002) on the time spent in immobility, as illustrated in Fig. 2D (Left panel). Males spent more time immobile on day 2. Moreover, mutant males spent more time immobile on days 1 and 2 as shown by post hoc analyses (+229% versus controls on day 1, +66% versus controls on day 2). Two-way ANOVA also showed an effect of day (F_(1, 25)_ = 5.92, p = 0.022) and genotype (F_(1, 25)_ = 6,38, p = 0.018) on the time spent climbing (Fig. 2D; Middle panel), with a reduced behavior for mutant males in particular during day 1 (−73% versus controls). Effects of day (F_(1, 25)_ = 26.90, p < 0.0001) and genotype (F_(1, 25)_ = 12.30, p = 0.0017) were also observed on the time spent in swimming (Fig. 2D; Right panel). Post hoc analyses showed less time spent in this behavior by mutant males in days 1 and day 2 (−27% and −36% versus controls, respectively). Therefore, when mutant males were exposed to stress, they showed increased despair-like behavior as evidenced by the increased time spent in immobility and conversely decreased time spent in climbing and swimming.

Altogether, these data indicate that under basal conditions, ERβ^NesCre^ males exhibited an unchanged anxiety-state level in the dark-light box and open-field, and a reduced anxiety-state level in the elevated O-maze. However, when exposed to unescapable stress, they exhibited a higher level of despair-like state compared to their control littermates.

### Effects of neural *ERβ* deletion on sucrose preference

**Figure 3:**
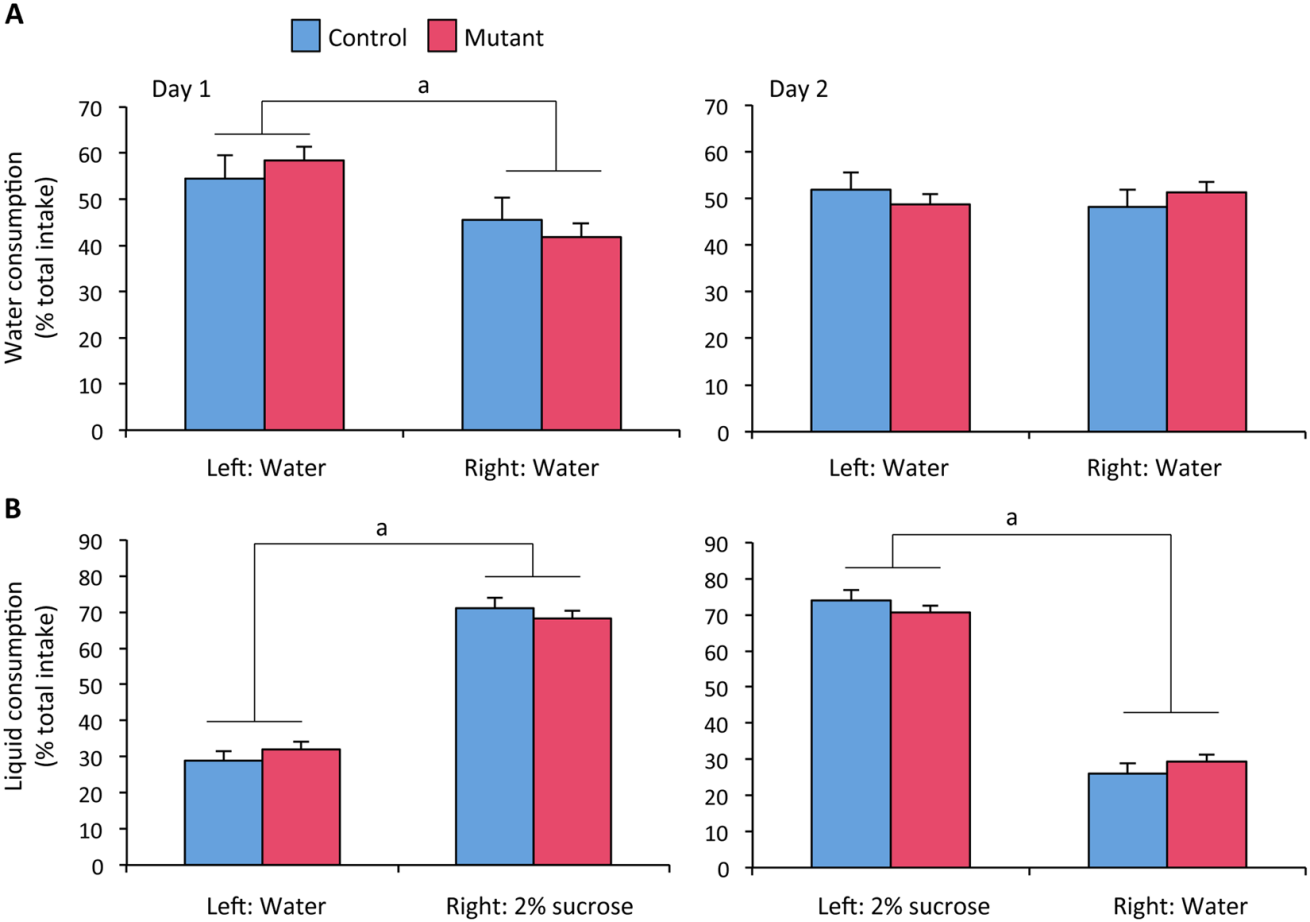
Effects of neural *ERβ* knockout on sucrose preference in male mice. Control and mutant males (n = 11 per genotype) were subjected to sucrose preference test with the habituation phase (**A**) and testing sessions (**B**). (**A**) Water consumption over two consecutive days 1 and 2. Two-way ANOVA shows an effect of location (^a^p < 0. 05) at day 1 but not at day 2. (**B**) Liquid consumption with the bottle containing 2% sucrose either at the right or left position. Two-way ANOVA shows an effect of solution for the two tests (^a^p < 0.001), with a preference of sucrose regardless of the genotype.

The consistency of the observed behavioral despair phenotype was further verified by analyzing a second group of mice (n = 11 per genotype) using the tail suspension test. In this test, mutant males spent more time in immobility (+35%) than their control littermates did (148 ± 9 sec for mutants *versus* 110 ± 9 sec for controls; p < 0.01). Other alterations that can be indicative of a depressed-like state were further assessed by evaluating anhedonia through sucrose consumption. Males were first habituated to this paradigm for two days, with the left and right drinking bottles containing water (Fig. 3A). On day 1, two-way ANOVA showed no effect of genotype (F_(1, 20)_ = 0.64, p = 0.43), but there was an effect of bottle location (F_(1, 20)_ = 6.14, p = 0.02). This preference for the left water bottle during day 1 disappeared during the second day since there was no longer an effect of bottle location (F_(1, 20)_ = 0.00, p = 0.98), with still an absence of genotype effect (F_(1, 20)_ = 2.51, p = 0.13). During the following days, one of the two bottles contained 2% sucrose and its location was changed on alternate days. Figure 3B shows that males exhibited increased consumption of sucrose solution whatever the location of the bottle containing this solution. Two-way ANOVA showed an effect of solution (F_(1, 20)_ = 83.95, p < 0.0001 in Test 1; F_(1, 20)_ = 25.36, p < 0.0001 in Test 2), but not of genotype (F_(1, 20)_ = 0.06, p = 0.81 in Test 1; (F_(1, 20)_ = 0.22, p = 0.64) in Test 2).

Altogether, these data strongly suggest that neural *ERβ* knockout males exhibit an increased despair-like behavior and normal sucrose preference.

### Neural *ERβ* deletion reduces aggressive behavior

**Figure 4:**
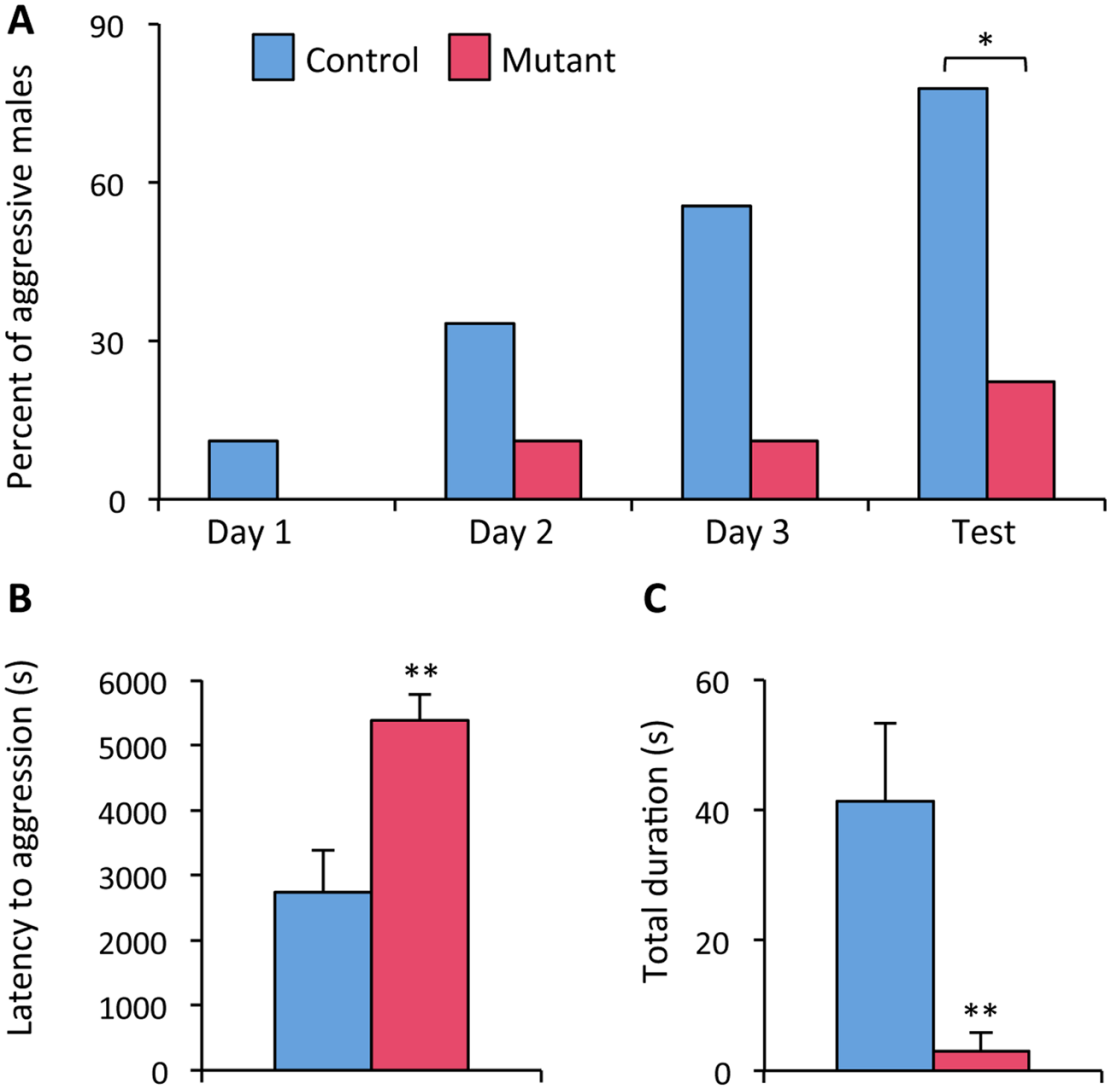
Aggressive behavior in control and mutant males. (**A**) Percentage of males (n = 9 males per genotype) exhibiting aggressive behavior towards intruder males over four consecutive days. ^*^p < 0.05 versus controls. (**B**) Latency to the first attack at Test day; ^**^p < 0.01 versus controls. (**C**) Total duration of aggressive behavior at Test day; ^**^p < 0.01 versus controls.

Social interaction occurs in a variety of social contexts, including those related to reproduction such as mating (interaction between congeners of opposing sex) and territoriality or aggression (interaction between male congeners). In a previous work, we showed that neural *ERβ* mutation does not interfere with male olfactory preference towards receptive females or with copulatory behavior^7^. As ERβ^NesCre^ males exhibited a lower social interaction with male congeners, we asked whether they exhibited an altered aggressive behavior. To answer this question, a third group of naive control and mutant males was mated with receptive females in order to increase their olfactory preference and aggressive behavior as shown previously^25^. These mice were then isolated in their home-cage for two weeks before being tested in the resident-intruder test. Aggressive behavior toward intruders was analyzed for four consecutive days. Two-way ANOVA showed an effect of day (F_(3, 48)_ = 4.6, p = 0.006) and genotype (F_(1, 16)_ = 9.29, p = 0.007) on the number of males engaged in aggressive behaviors (Fig. 4A). Post hoc analysis showed a significantly increased number of aggressive males in the control group. This reached the fourth day of the test 78% of the total number of control males, while that of mutant males remained low (a maximum of 22%). In addition, the latency to exhibit the first aggressive behavior was significantly increased (+97% versus controls) in mutant males (Fig. 4B). Analysis of males that elicited aggressive behavior shows that the total duration of aggression towards an intruder was also significantly reduced in mutant males (−93% versus controls; Fig. 4C). Overall, the results indicate that mutant males exhibit a severely reduced aggressive behavior.

### Neural *ERβ* deletion does not alter corticosterone levels in males

**Figure 5:**
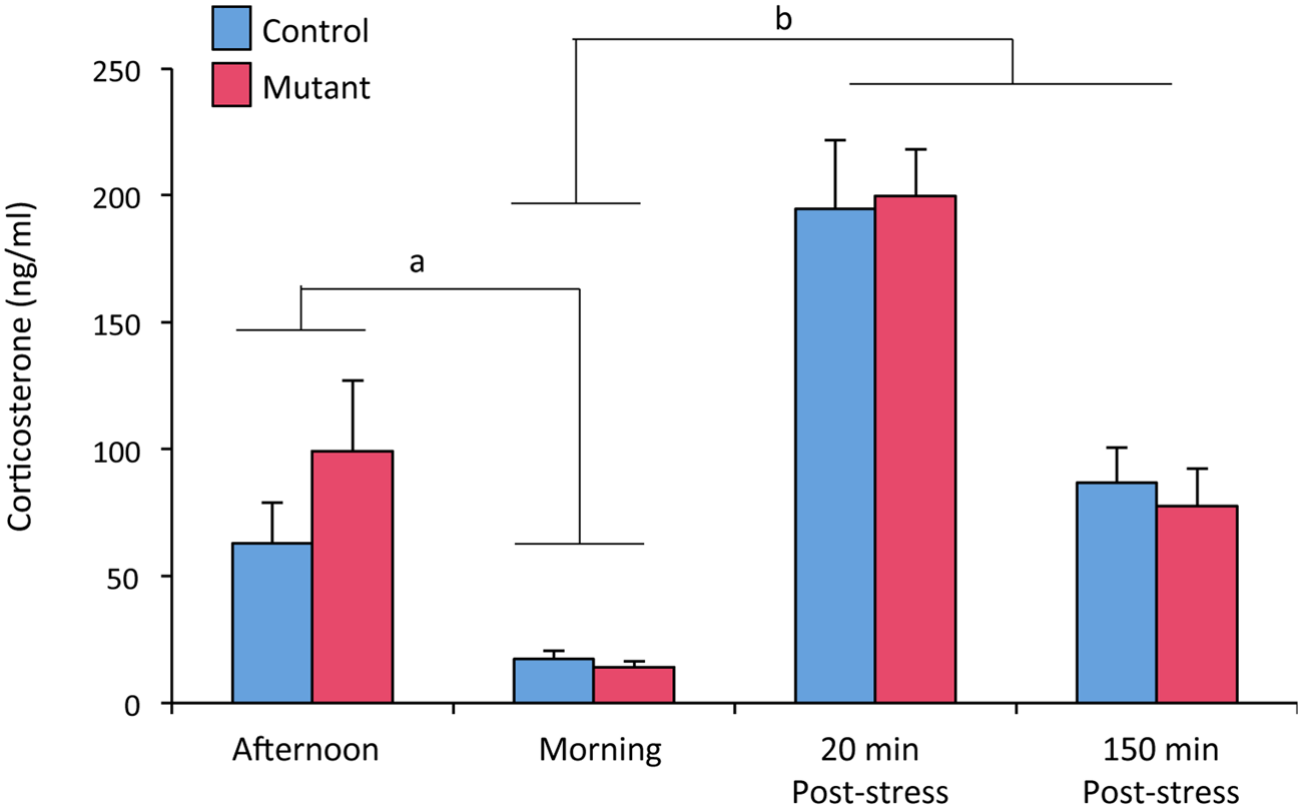
Corticosterone levels in control and mutant male mice. Corticosterone levels were measured in plasma samples collected in the afternoon and the following morning in control and mutant mice (n = 8–9 per genotype). They were then subjected to restraint stress and plasma was collected and measured for hormones 20 min and 150 min after the stress. ^a^p < 0. 001 versus afternoon levels; ^b^p < 0.001 versus morning levels.

The behavioral despair of neural *ERβ* knockout males could be due to changes in glucocorticoid levels since ERβ was previously reported to regulate the hypothalamus-pituitary-adrenal (HPA) axis^26^. An assessment of circulating corticosterone levels was performed in both basal conditions and after stress (Fig. 5). In basal conditions, there was an effect of time (F_(1, 15)_ = 15.99, p = 0.0012) but not of genotype (F_(1, 15)_ = 0.95, p = 0.344). As expected, corticosterone levels were higher at the end of the afternoon in comparison with the morning for both controls and mutants (4- to 7- fold above the levels at 9:00 am, respectively). There was also an effect of stress (F_(2, 30)_ = 66.24, p < 0.0001) but not of genotype (F_(1, 15)_ = 0.03, p = 0.855). Corticosterone levels were increased by 11-fold in controls and 14-fold in mutants at 20 min post-stress, compared to basal levels at 9:00 am. They were then decreased by 55% in controls and 61% in mutants at 150 min post-stress. Altogether, these data show that there was no significant alteration of the HPA axis integrity in neural *ERβ* knockout males.

### Effects of neural *ERβ* deletion on female behaviors and number of TPH2-immunoreactive neurons in male and female mice

Previous studies reported that ubiquitous *ERβ* deletion increases anxiety- and depressive-like behaviors in female mice, and that these effects involve modifications in serotonergic neurons with reduced TPH2-immunoreactivity in the dorsal raphe^24^. Thus, we first asked whether the conditional ERβ^NesCre^ mutation triggers a similar phenotype in females, and second if the neural behavioral changes observed in neural *ERβ* knockout males can also be associated with modifications in the number of TPH2-immunoreactive neurons.

**Figure 6:**
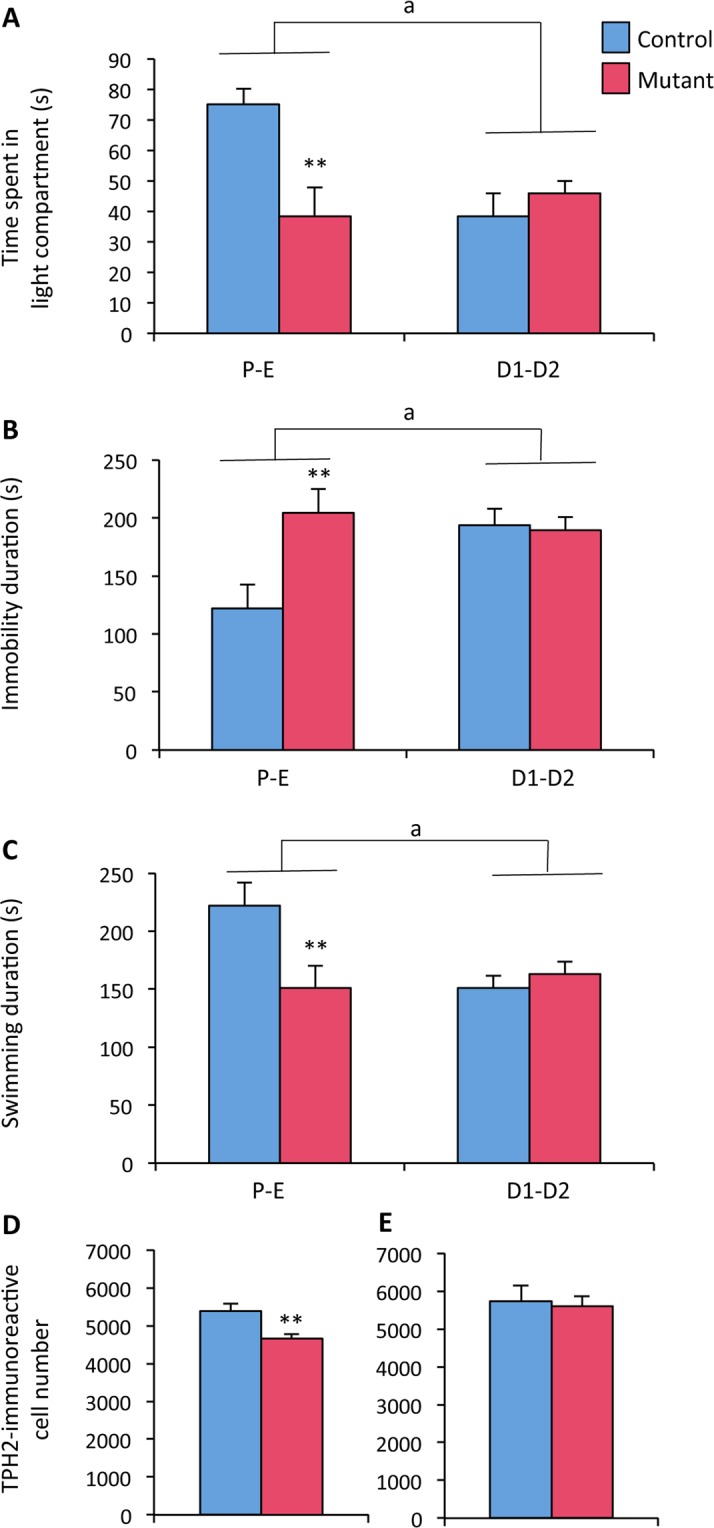
Anxiety- and despair-like behaviors in females, and TPH2 immunoreactivity in males and females. (**A**) Time spent in the light compartment of the dark-light box by control and mutant females at the proestrous/estrous (PE-E) or diestrous (D1-D2) stage (n = 18–29 females per genotype). Two-way ANOVA shows an effect of cycle (^a^p < 0.01); posthoc analyses indicate a reduced time spent in the light compartment for mutant females at the P-E stage (p < 0.01 versus controls at the same stage). (**B**) Time spent in immobility in the forced swim test for females at the P-E or D1-D2) stage. Two-way ANOVA shows an effect of cycle (^a^p < 0.05); posthoc an^a^lyses indicate an increased time spent in immobility for mutant females at the P-E stage (p < 0.01 versus controls at the same stage). (**C**) Time spent swimming in the forced swim test for females at the P-E or D1-D2) stage. Two-way ANOVA shows an effect of cycle (^a^p < 0.05); posthoc analyses indicate a reduced time spent in swimming for mutant females at the P-E stage (p < 0.01 versus controls at the same stage). (**D,E**) Number of TPH2 neurons counted in the dorsal raphe of females (**D**) and males (**E**). Data are means ± S.E.M. of 6–8 animals per sex and per genotype; ^**^p < 0.01 versus controls.

In behavioral analyses performed on females, the changes in estrogen levels during the estrous cycle were taken into account by dividing the females into two groups. One group contained females in pro-estrous and estrous phases (high estrogen levels) and the second one contained females in diestrous phases (low estrogen levels). The anxiety state level was analyzed in the dark-light box (Fig. 6A). Two-way ANOVA showed an effect of estrous cycle (F_(1, 43)_ = 4.93, p = 0.003) and genotype (F_(1, 43)_ = 4.92, p = 0.03). Control females of the diestrous group spent less time in the light compartment than females of the proestrous-estrous group (−49%). Furthermore, mutant females at the proestrous/estrous phase spent a lower time in the light compartment compared to their control littermates at the same estrous phase (−50%; p < 0.01). Therefore, the estrogen-induced anxiolytic effect observed during the proestrous/estrous phase disappeared in neural *ERβ* knockout females. This result is in agreement with our previous analysis using the O-maze test^6^, indicating that the neural ERβ mediates estrogen-anxiolytic effects during the pro-estrous/estrous phase.

In the forced swim test, two-way ANOVA showed an effect of the estrous cycle (F_(1, 62)_ = 3.89, p < 0.05) and genotype (F_(1, 62)_ = 6.95, p = 0.04) on the time spent in immobility (Fig. 6B). Post hoc analysis showed that mutant females spent an increased time spent in immobility at the proestrous/estrous phase (+60% versus control littermates at the same estrous phase). Effects of the estrous cycle (F_(1, 62)_ = 4.01, p = 0.04) and genotype (F_(1, 62)_= 4.27, p = 0.01) were also observed on swimming behavior (Fig. 6C), with less time being spent in this behavior by mutant females at the proestrous/estrous phase (−32% versus controls). Altogether, these data indicate that the phenotype previously reported in the total ERβKO model must be mainly caused by the lack of the neural ERβ.

We next investigated the effects of neural *ERβ* knockout on the number of TPH2-immunoreactive neurons in the dorsal raphe of both males and females. Quantification by immunohistochemistry was performed in the dorsal raphe of females at the proestrous/estrous phase and in males. Data illustrated in Fig. 6D show a significant reduction of the TPH2-immunoreactive cell number in mutant females (−12% versus controls). In contrast, no significant difference was observed in the number of TPH2-immunoreactive cells in the dorsal raphe between controls and mutant males (Fig. 6E).

### Neural *ERβ* deletion lowers arginine-vasopressin and oxytocin expression levels in the BNST

**Figure 7:**
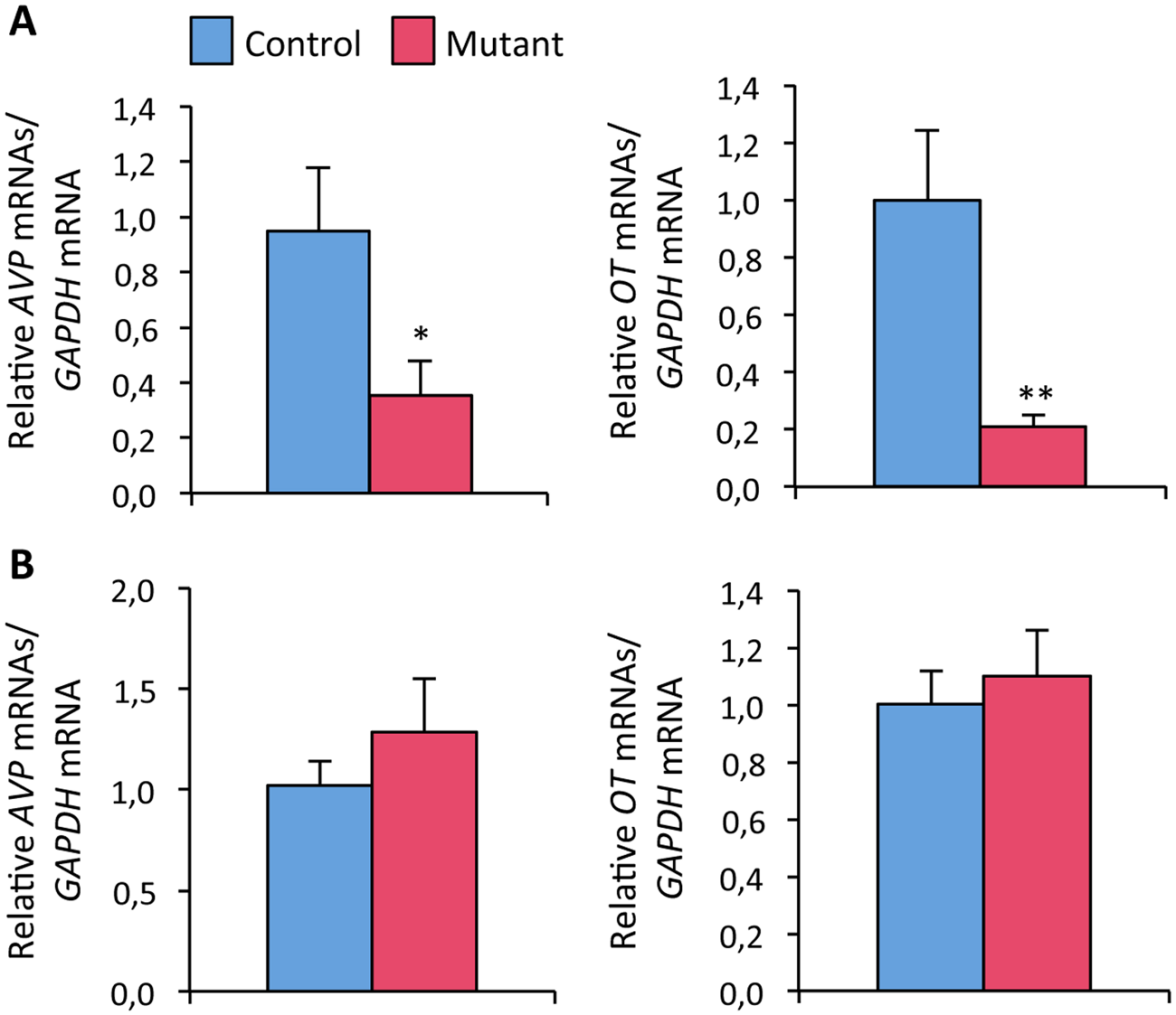
Effects of neural *ERβ* deletion on *OT* and *AVP* expression in male mice. (**A**) Levels of *AVP* (*Left*) and *OT* mRNAs (*Right*) normalized to *GAPDH* expression in the bed nucleus of stria terminalis of males (n = 9 animals per genotype). ^*^p < 0.05 or ^**^p < 0.01 versus controls. (**B**) Levels of *AVP* (Left) and *OT* (Right) expression normalized to *GAPDH* in the hypothalamic paraventricular nucleus.

The behavioral modifications (increased behavioral despair, reduced social and aggressive behaviors) triggered by neural *ERβ* deletion cannot be due to changes in circulating levels of testosterone since mutant and control males exhibit similar hormonal levels^7^. Mutant males also present normal HPA integrity and TPH2-immunoreactivity in the dorsal raphe. We thus investigated whether the observed behavioral modifications could be related to changes in the expression levels of AVP and OT, two neuropeptides also involved in the regulation of social and mood-related behaviors in two key regions i.e. the BNST and paraventricular nucleus^18,26–28^. Levels of *AVP* and *OT* mRNAs were quantified by RT-qPCR and normalized to GAPDH levels in control and mutant males. Figure 7A shows that *AVP* and *OT* expression levels were significantly reduced in the BNST of mutant males (−63% and −79% *versus* controls, respectively). In contrast, no differences were seen in the expression levels of *AVP* and *OT* measured in the paraventricular nucleus (Fig. 7B).

## Discussion

The present study shows that neural *ERβ* deletion in male mice increases despair-like behavior, reduces social interaction and impairs aggressive behavior towards same-sex congeners. These behavioral alterations were associated with changes of *OT* and *AVP* expression in the BNST. Expression levels of these neuropeptides in the paraventricular nucleus, circulating levels of corticosterone and number of TPH2-immunoreactivity in the dorsal raphe were not modified in mutant male mice.

Social behavior was assessed in control and mutant mice by using several tests. In the three-chamber paradigm analyzing interest in a same-sex congener versus an empty box, no significant differences were observed in time spent in the compartments or in olfactory investigation between the two genotypes. In contrast, in the second paradigm evaluating social interaction with a freely moving same-sex congener, mutant males spent less time in investigation by comparison to their control littermates. In accordance with this result, a low percentage of mutant males (22%) exhibited aggressive behavior towards non-aggressive intruder males in the resident-intruder test. The majority of tested mutant males (78%) did not display any behavior over the 4-days test. These behavioral modifications were not due to a reduced locomotor activity. Indeed, mutant males were even more active than males of the control group in the circular corridor test. It is interesting that when tested for mating behavior, mutant males showed comparable olfactory preference towards females, and latencies to initiate mounting and thrusts and reach ejaculation in comparison to their control littermates^7^. The number of behavioral events during the copulatory phase was also similar between the two genotypes. Altogether, these data indicate that neural *ERβ* deletion selectively impairs social interaction and aggressive behavior towards same-sex congeners, without altering mating behavior.

Mating and aggressive behaviors are stimulated by olfactory cues, which activate chemosensory regions such as the medial amygdala, then the BNST. Chemosensory signals are transmitted to the medial preoptic area for sexual behavior, and to other structures including lateral septum and ventromedial nucleus for aggression. Previous studies using global aromatase or *ERα* knockouts, or neural deletion of the *androgen receptor* (*AR)* showed severely impaired expression of aggressive behavior in male mice^8,25,29,30^, indicating that both estrogen- and androgen-dependent signaling pathways regulate this male behavior. The present study shows that ERβ is also involved and points out possible cross-regulations between these signaling pathways, at least partly in specific brain areas such as the BNST. Indeed, our previous analyses^7^ showed that the number of AR- and ERα-immunoreactive neurons is modified in the BNST, but not in the medial amygdala or preoptic area of mutant males lacking the neural *ERβ*. This suggests a particular role of the BNST, a brain structure, situated at the intersection of several key circuits central to social and reproductive behaviors, mood-related behaviors, and regulation of the hypothalamus pituitary adrenal (HPA) axis^27,31^.

The present experiments using different conflict paradigms show that neural *ERβ* mutation in mice results in unchanged anxiety level in the dark light-box and open-field tests, and reduced behavior basal anxiety level in the elevated O-maze. This latter anxiolytic-like effect of the mutation could be related to the increased activity of mutant males as measured in the circular corridor. A previous mouse study reported minor effects of global *ERβ* knockout on the anxiety state level measured with the open-field and elevated plus maze tests in male mice^16^. By contrast, chronic administration of androgen metabolites with actions at ERβ to castrated male rats decreased the anxiety state level in the elevated plus maze and dark-light box^19^. Another rat study showed that acute treatment of castrated males with a selective *ERβ* agonist reduced anxiety-like behavior and corticosterone and ACTH responses to stress^18^. It was concluded by the authors that ERβ modulates the display of anxiety-like behaviors and HPA reactivity in rats. In male mice, our data did not show any impact of neural *ERβ* deletion on the integrity of the HPA axis either under basal conditions or following an induced restraint stress. This apparent discrepancy between male mice and rats could be due, at least partly, to the recently reported chemoarchitectural differences in ERβ neurons of the paraventricular nucleus between the two species^32^. Indeed, only a modest number of CRH-cre containing cells were found to express ERβ in male mice by comparison to rats.

When ERβ^NesCre^ males were exposed to an inescapable stress, using the forced swim and tail suspension tests classically used to evaluate behavioral despair, they showed a marked increase in immobility time compared with control littermates. Interestingly, this increased despair-like behavior was associated with normal sucrose preference. Depression is generally characterized by several facets including social aversion, anxiety, resignation and anhedonia. ERβ^NesCre^ males exhibited part of, but not the whole, depressive-like pattern since they displayed only reduced social interaction and increased despair-like behavior. This suggests that they may present a mild depressed-like state. In similar experimental conditions, and in contrast to males, neural knockout *ERβ* females showed both an increased anxiety-state level and despair-like behavior, as well as a significantly reduced number of TPH2-immunoreactive neurons in the dorsal raphe, confirming previous data obtained on global *ERβ* knockout females^24^. Therefore, these observations point out the existence of sex differences in the effects triggered by neural *ERβ* mutation on anxiety- and despair-like behaviors. As all our behavioral tests were conducted on 2–4-month old males, it would be interesting to investigate whether the observed mood phenotype is maintained or exacerbated in middle and late aged males.

Several studies reported ERβ-mediated regulation of AVP and OT expression in the medial amygdala and paraventricular nucleus of male rats^33–36^. Nevertheless, few studies are available for mice. A previous study showed that estrogen treatment increased OT and decreased AVP expression in the paraventricular of wild type males, and this regulation was abolished in global βERKO mice^11^. Another study showed that mRNAs levels of OT and AVP were correlated with ERβ mRNAs in the paraventricular nucleus of male mice, while the expression levels of their receptors were better correlated with ERα mRNAs levels in the medial amygdala^28^. The present study shows that *ERβ* deletion in neuronal and glial progenitors targeted by the NesCre transgene triggers different effects on *AVP* and *OT* expression in the BNST and paraventricular nucleus. In particular, a significant reduction in *OT* and *AVP* expression was observed in the male BNST. As mentioned above, this brain area is located at the intersection of several key circuits underlying social and mood-related behaviors and involving OT and AVP neuropeptides. For instance, infusions of the V1a receptor antagonist into the medioventral BNST of California mice induced anxiogenic effects in social and nonsocial contexts^37^. Blocking the OT receptor in the dorsolateral BNST reduced the acquisition of conditioned cued fear, but left the baseline startle and non-cued fear (background anxiety) intact^38^. Moreover, the ablation of AVP cells in the BNST of male mice reduced social investigation of other males, and increased their sucrose intake^39,40^. Indeed, the BNST receives OT and AVP projections from the paraventricular nucleus, but it also contains AVP and OT neurons. The unchanged mRNA levels for the two neuropeptides in the paraventricular nucleus, and reduced expression in the BNST, led us to suggest that the behavioral alterations induced by neural *ERβ* mutation could be related at least in part to modifications in AVP and OT neurons of the BNST, although the involvement of other brain areas cannot be excluded. The BNST is a sexually dimorphic structure and target of sex steroid hormones during the perinatal surge of testosterone in males. Whether or not ERβ is important perinatally in the organization of this structure or rather later remains to be assessed. Previous studies showed that estrogens through both ERα and ERβ are required in the whole organization of this brain region in male mice^41^. Further studies are needed to investigate the behavioral effects of restricted *ERβ* deletion in the BNST.

In conclusion, we show that neural *ERβ* deletion in male mice triggers increased despair-like behavior, with no anhedonia or increased anxiety-like behavior. It also interferes greatly with the expression of social behaviors directed towards same-sex congeners (social interaction, aggressive behavior). This suggests that ERβ signaling pathway is involved, to different extent levels, in mood-related behaviors and social interaction with same-sex congeners. The mild depressive-like behavior was associated with unchanged TPH2-immunoreactivity in the dorsal raphe, thereby highlighting sex differences as evidenced by increased anxiety- and despair-like behaviors and reduced TPH2-immunoreactivity in neural *ERβ* knockout females. The integrity of the HPA axis and OT and AVP mRNAs in the paraventricular nucleus were unaffected, while changes were seen in the mRNAs levels of AVP and OT in the BNST of male mice. Altogether, these data document the neural function of ERβ in male mice with respect to estrogen-induced modulation of mood-related and social behaviors, and suggest a potential role for the ERβ signalling pathway in the BNST. Further gene deletion restricted to this brain area could help to confirm these observations.

## Methods

### Animals

The ERβ^NesCre^ mouse line was obtained on a C57BL/6J genetic background, as previously described^6,7^. Control mice (ERβ^fl/fl^) and their mutant littermates (ERβ^fl/fl^ carrying the NesCre transgene; ERβ^NesCre^) were group-housed under a controlled photoperiod (12:12 h light–dark cycle – lights on at 7 am), maintained at 22 °C, with free access to food and water. All studies were carried out on 2–4 months old animals, in accordance with the European legal requirements (Decree 2010/63/UE) and were approved by the “Charles Darwin” Ethical committee (project number 01490-01). For females, vaginal smears were taken for two weeks before the behavioral and immunohistochemical analyses in order to determine the stage of the estrous cycle on the day of experiment. Depending on the result, females were separated into two groups: those in proestrous/estrous (high estradiol levels) or diestrous (low estradiol levels).

### Behavioral tests

Three groups of males were analyzed. The first was subjected to tests of social interaction, locomotor activity, anxiety-state level (O-maze and dark-light box), and forced swim test. A second was analyzed in the tail suspension and sucrose preference tests. Finally, the third one was analyzed in the resident-intruder test after a first sexual experience.

#### Sociability

The sociability of mice was analyzed as previously described^42^. The apparatus was a rectangular three-chambered box (400 mm W × 225 mm H × 600 L: 200 mm each chamber). Habituation: the test mouse was first placed in the middle chamber and allowed to explore for 10 min, with the doorways into the two side chambers open. Each of the two side rooms contained an empty wire corral. After the habituation period, the test mouse was enclosed in the middle room of the box, and an unfamiliar male mouse was enclosed in one of the corrals placed in a side chamber. The doors were then opened, and the test mouse was allowed to explore the entire social box for 10 min. The time spent by the test mouse in chambers and the number of entries into each chamber were measured. The time spent by the test mouse investigating the stimulus mouse was also recorded.

#### Social interaction

This was evaluated according to our previous protocol^42^. Social interaction was tested in a cage (155 mm W × 266 mm H × 425 mm L), the floor of which was covered with an 0.5 cm layer of clean bedding. The test mouse and an age- and sex-matched C57Bl/6 J congener were individually housed in a standard mouse cage for 1 h prior to the test session, before being introduced in the test cage. Interactions were recorded for 10 min. Time spent by the test mouse in investigation, including anogenital and nose-to-nose sniffing, was subsequently scored from digital videotapes.

#### Locomotor activity

Activity of animals was analyzed in a computed circular corridor as previously described^6^. Briefly, the subject male was introduced into a circular corridor made of two concentric cylinders crossed by four diametrically opposite infrared beams (Imetronic). Locomotor activity was counted when animals interrupted two successive beams and had thus traveled a quarter of the way around circular corridor. The locomotor activity test lasted 140 min.

#### Anxiety-related behavior

The elevated zero maze test was conducted as previously described^6^. Males were placed in the closed arms and were allowed to explore the maze freely for 9 min. The number of entries and the time spent in the open arms were analyzed. A mouse was considered to be in an open arm when all its 4 paws had entered. The light intensity was 60 lux.

The dark-light box (200 mm W × 200 mm H × 450 mm L) consisted of two compartments that communicate through an opening of 7.5 × 7.5 cm. The dark compartment (200 mm W × 200 mm H × 150 mm L) had a cover and black walls, whereas the uncovered lighted room (200 mm W × 200 mm H × 300 mm L) had white walls lit up to approximately 800 lux. The mouse was placed in the center of the dark compartment at the beginning of the test. The number of entries and total time spent in the light compartment during the 5 min test were measured.

The open-field consisted of a white Plexiglas field (430 mm W × 250 mm H × 430 mm L). The time spent in the center zone and periphery was scored for 9 min. This test was conducted on a fourth group of males as presented in Supplemental Fig. 1.

#### Forced swim and tail suspension tests

For the forced swim test, animals were placed individually in a glass beaker filled with 25 ± 1 °C tap water to a depth of 12 cm. The same experiment was repeated the following day to assess the effect of habituation. Each session was recorded for 6 min, and the obtained videotapes were scored afterward in order to measure the time spent in immobility, swimming, and climbing.

In the tail suspension test, the mice tails were suspended with tape and their behavior was recorded for 6 min. The total immobilization time was measured.

#### Sucrose preference

The sucrose preference test consisted of a two-bottle choice paradigm. Mice were first habituated to two bottles of water for 48 h. They were then given access to two pre-weighed bottles: one containing water and the other one 2% sucrose, for 4 days. The position of the two bottles was alternated every 24 h in order to discriminate between a positional preference and sucrose preference. Bottles were weighed every day to measure water and sucrose consumption.

#### Resident-intruder test

Males of both genotypes were individually housed for 3 days. Each male was then paired in its home cage with a sexually receptive female and allowed to reach ejaculation. Receptive C57BL/6 J females were ovariectomized, supplemented with implants containing estradiol-benzoate (Sigma-Aldrich) and primed with progesterone (Sigma-Aldrich) 4–5 h before the test as previously described^8^. This first sexual experience was performed in order to increase the expression of male behaviors such as olfactory preference and aggression as previously described^25^. Males were then left in their home cage without bedding change for 2 weeks before starting analyses. Aggressive behavior of residents was analyzed for 10 min each day, through four consecutive days. Experiments took place 2 h after lights were turned off and began when the intruder, an adult A/J male mouse (The Jackson Laboratory), was introduced into the home cage of the resident test mouse. Each resident was tested with a new intruder each day. Aggression was defined as lunging, biting and wrestling. For each animal, the latency to attack and the total duration of aggressive episodes were measured. The latency to attack was set to 600 s if the resident showed no aggressive behavior.

### Hormonal levels

Corticosterone levels were monitored under basal conditions in the afternoon (6 pm) by collecting blood from the tail vein into heparinized capillary tubes. The following day, blood from the same animals was collected in the morning (9 am). The test animals were then restrained for 20 min in well-ventilated 50-ml tubes, before being replaced in their home cage. Samples, collected at the end of restraint stress and 150 min later, were frozen until the assay. Corticosterone levels were determined with a radioimmunoassay kit (MP Biomedicals, #07-120102), with inter- and intra-assay coefficients of variation of 7.1% and 4.4%, respectively.

### TPH2 immunohistochemistry

Animals were perfused transcardially with a solution of 4% paraformaldehyde (PFA) in phosphate buffer after blood removal with 0.9% NaCl. After overnight post-fixation at 4 °C with 4% PFA and cryoprotection for two days in 30% sucrose solution, brains were frozen at −30 °C in isopentane and kept at −80 °C before processing in a cryostat. Coronal sections of 30 μm comprising the dorsal raphe nucleus were harvested on Superfrost plus glass plates. After saturation in phosphate buffer saline (PBS), triton X-100 0.2% and BSA 1% for 2 h at room temperature, sections were incubated in blocking buffer with primary polyclonal anti-TPH2 (1/500; Novus Biologicals) overnight at 4 °C. After 3 washes with PBS, incubation with secondary biotinylated goat anti-rabbit (1:500; Vector Laboratories) was performed for 2 h at room temperature. Revelation was carried out using the streptavidin complex reagent (Vector Laboratories), followed by color development with the 3,3-diaminobenzidine tetrahydrochloride chromogenic substrate (Sigma–Aldrich). Mounted sections were scanned with a Hamamatsu Nanozoomer (Institut de la Vision, Paris) and TPH2-expressing neurons were manually counted, using NDPview software, in anatomically matched sections of the dorsal raphe (plates 64-75 of the Mouse Brain Atlas of Paxinos and Franklin;^43^).

### Quantitative RT-PCR

Tissue punches were recovered through the bed nucleus of stria terminalis (BNST) and paraventricular nucleus with a 1 mm diameter canula from 400 μm thick brain slices corresponding, respectively, to plates 29–31 and 36–39 of the Mouse Brain Atlas^43^. They were rapidly frozen at −80 °C until use. Total RNAs were extracted from tissues using the PicoPure RNA isolation kit (Excilone, Vicq, France). RNA (120 ng) was reverse transcribed into cDNA using the Superscript III first Strand Synthesis System (Invitrogen) with random hexamers. Real-time PCR was carried out in a LightCycler 480 II (Roche, Mannheim, Germany) using LightCycler 480 SYBR Green I PCR Mastermix (Roche) as previously described^44^.

Each DNA sample was analyzed in triplicate along with standard and no-template controls. The incubation media contained 2.5 µl of each reverse transcription reaction in 10 µl of Mastermix, along with 200 nM forward and reverse primers. *OT* primers (AmplifX for forward *OT* primer: TGC TTG GCT TAC TGG CTC TGA; reverse *OT* primer: CAG GGG AGACAC TTG CGC ATA), *AVP* primers (St Louis *et al*., 2012; forward AVP primer: GCG GCA AGA GGG CCA TCT CTG AC, reverse AVP primer: TCC GCG CAG CAG ATG CTT GG TC), and primers for the glyceraldehyde 3-phosphate dehydrogenase (GAPDH) gene used as an internal control (Gilsbach *et al*., 2006; forward *GAPDH* primer: TGC-ACC-ACC-AAC-TGC-TTA-GC, reverse *GAPDH* primer: GGC-ATG-GAC-TGT-GGT-CAT-GAG) were synthesized (Eurogentec, Liège, Belgium) and their specificity was checked using NCBI GenBank. PCR parameters were 95 °C for 5 min, then 45 cycles of 95 °C for 15 sec, 61 °C for 15 sec, and 72 °C for 10 sec.

PCR specificity was verified by melting curve analysis and product purity was confirmed by agarose gel electrophoresis. All experiments had efficiencies between 96 and 105% and were analyzed with the LCS 480 version 1.5 software (Roche). Data from each sample run in triplicate allowed an average cycle threshold (Ct) value to be obtained, and the relative expression of each target gene was determined using the comparative Ct method.

### Statistical analyses

Data were expressed as means ± S.E.M. Normality tests (Kolmogorov-Smirnov and Shapiro-Wilks tests) were performed before using the parametric analyses. Two-way ANOVA was used to analyze the main effects of genotype and compartment for sociability, genotype and time for locomotor activity, genotype and day for forced swim test and aggression, genotype and bottle location or solution for sucrose preference test, genotype and time or stress for glucocorticoid measurements, genotype and estrous cycle for female behaviors. Bonferroni post-hoc tests were used to determine group differences. Student’s t-tests were used to determine the effect of neural *ERβ* deletion for the remaining data. P values of less than 0.05 were considered to be significant.

## Supplementary information

Supplementary information 

## Data Availability

The datasets generated during and/or analyzed during the current study are available from the corresponding author on reasonable request.
