# Supplementary material for: Effects of neural estrogen receptor beta deletion on social and mood-related behaviors and underlying mechanisms in male mice
Source: Sci Rep. 2020 Apr 10;10:6242. doi: 10.1038/s41598-020-63427-4 (PMC7148327; doi:10.1038/s41598-020-63427-4)
Supplement: Supplementary file 1 — Supplementary information [file 41598_2020_63427_MOESM1_ESM.docx]

**Effects of neural estrogen receptor beta deletion on social and mood-related behaviors and underlying mechanisms in male mice**

Carlos Dombret, Lydie Naulé, Anne-Charlotte Trouillet, Caroline Parmentier, Hélène Hardin-Pouzet, Sakina Mhaouty-Kodja

**Supplemental Figure 1.** The open-field test was used on a fourth group of males. Time spent in the center and periphery of the open-field by control and mutant males (n = 8-9 per genotype). There was an effect of location (F_(1, 15)_ = 853.6, ^a^p < 0.0001) but not of genotype (F_(1, 15)_ = 3.205, p = 0.1).
